# Potential Neuroprotective Role of GLP-2 in Alzheimer’s Disease: Clinical Observations, Mechanistic Insights, and Comparison with GLP-1

**DOI:** 10.3390/ijms27031609

**Published:** 2026-02-06

**Authors:** Maciej Czarnecki, Agnieszka Baranowska-Bik, Anna Litwiniuk, Małgorzata Kalisz, Anita Domańska, Anna Kurdyła, Wojciech Bik

**Affiliations:** 1 Neuroprotect Medical Center, 01-684 Warsaw, Poland; maciej.czarnecki@neuroprotect.pl (M.C.); anna.kurdyla@neuroprotect.pl (A.K.); 2 Department of Endocrinology, Centre of Postgraduate Medical Education, 01-813 Warsaw, Poland; 3 Department of Neuroendocrinology, Centre of Postgraduate Medical Education, 01-813 Warsaw, Poland; alitwiniuk@cmkp.edu.pl (A.L.); mkalisz@cmkp.edu.pl (M.K.); wojciech.bik@cmkp.edu.pl (W.B.)

**Keywords:** glucagon-like peptide 2 (GLP-2), Alzheimer’s disease (AD), dementia

## Abstract

Alzheimer’s disease (AD) is the most common cause of dementia and is characterized by progressive cognitive decline, β-amyloid accumulation, tau pathology, oxidative stress, and neuroinflammation. Increasing evidence suggests that metabolic dysregulation may contribute to AD pathogenesis. Glucagon-like peptide-2 (GLP-2), an intestinal peptide hormone, has demonstrated neuroprotective effects in preclinical models, potentially through anti-inflammatory and anti-apoptotic mechanisms. However, its role in human neurodegenerative disorders remains insufficiently understood. This study aimed to compare plasma GLP-2 concentrations between individuals with AD and cognitively healthy controls and to examine associations between GLP-2 levels, cognitive impairment severity, and metabolic parameters. Sixty-one patients with clinically diagnosed AD and twenty-three cognitively unimpaired controls were recruited. Plasma total GLP-2 concentrations were assessed at baseline in all participants and additionally at 6 and 12 months in a subgroup of 34 AD patients. Cognitive function was evaluated using the Mini-Mental State Examination (MMSE) and the Clinical Dementia Rating (CDR) scale. Group comparisons, subgroup analyses based on AD severity, repeated-measures analyses, Spearman correlations, and multivariable linear regression models (including age and clinical group) were performed. Plasma GLP-2 concentrations were significantly higher in AD patients than in controls, with a moderate effect size (Cohen’s d ≈ 0.60). In severity-based subgroup analyses, both the mild and moderate-to-severe AD groups showed significantly higher GLP-2 levels than controls. Longitudinal analyses in AD patients (n = 34) showed no significant changes in GLP-2 concentrations over 12 months. Cognitive performance declined over time, with a significant reduction in MMSE from baseline to 6 months, whereas GLP-2 levels were not correlated with MMSE or CDR at any time point. GLP-2 levels correlated positively with body mass index (BMI), body weight, insulin, and HOMA-IR. In multivariable regression analysis, neither age nor clinical group independently predicted GLP-2 concentrations (both *p* > 0.05). Plasma GLP-2 concentrations were higher in patients with AD than in cognitively healthy controls; however, GLP-2 levels were not associated with cognitive performance or its progression over 12 months. GLP-2 was positively related to markers of adiposity and insulin resistance, suggesting stronger links to metabolic status than to cognitive severity. Further studies are needed to clarify whether GLP-2 alterations in AD reflect compensatory mechanisms, metabolic factors, or disease-related pathophysiology.

## 1. Introduction

Dementia syndromes are among the most significant public health concerns due to population aging. According to a study by Nichols et al. (2022), approximately 57.4 million people worldwide were living with dementia in 2019, with this number projected to rise to 152.8 million by 2050 [1]. Alzheimer’s disease (AD), an age-related neurodegenerative disorder, is estimated to account for 60–70% of all dementia cases and is the fifth leading cause of death among individuals over the age of 65 [2].

AD is characterized by two main pathological hallmarks: the accumulation of extracellular β-amyloid (Aβ) plaques and intracellular neurofibrillary tangles (NFTs), which are composed of hyperphosphorylated tau protein. These changes are strongly associated with synaptic and neuronal loss, dystrophic neurites, and gliosis, involving alterations in the morphology and function of microglia and astrocytes. Studies have shown that microglial overactivation in response to amyloid plaques increases the release of pro-inflammatory cytokines, which can damage neurons [3]. Oxidative stress, resulting from accumulating reactive oxygen species (ROS), is another key pathological feature of AD. Additionally, mitochondrial dysfunction leads to reduced ATP production, calcium dysregulation, and increased ROS generation. This dysfunction further exacerbates oxidative stress, creating a vicious cycle that accelerates neurodegeneration. Research has highlighted the role of oxidative stress and mitochondrial dysfunction in AD progression, as ROS contribute to Aβ plaque and tau tangle formation, ultimately leading to neuronal cell death [4,5]. Vascular changes also play an important role in AD pathogenesis. Studies have shown that cerebrovascular disease and AD often coexist, with vascular risk factors such as hypertension and diabetes accelerating disease progression [6].

All these pathological mechanisms contribute to synaptic degeneration, leading to progressive neurodegeneration and cognitive decline.

It is essential to highlight that hyperinsulinemia and insulin resistance (IR), key pathophysiological mechanisms of type 2 diabetes mellitus (T2DM), have been shown to impact cognitive function. IR, defined as reduced sensitivity to insulin, has been consistently identified as an essential risk factor for neurodegeneration and cognitive impairment [7,8]. AD is also linked to impaired insulin signaling in the brain, and T2DM patients have an increased risk of developing AD [9,10]. Therefore, ongoing research explores the influence of carbohydrate and insulin metabolism disorders on dementia-related processes.

Proglucagon is a precursor protein that gives rise to several biologically active peptides, some of which play crucial roles in regulating glucose homeostasis, insulin secretion, and appetite control. The proglucagon family comprises various peptides derived from the proglucagon protein, the most well-known of which are glucagon and glucagon-like peptides (GLP-1 and GLP-2). The proglucagon gene is expressed in multiple tissues, notably the pancreas, intestine, and brain, producing peptides with diverse physiological effects [11].

Notably, patients with type 2 diabetes treated with semaglutide had a lower risk of developing AD compared to those receiving other diabetes therapies [12]. Additionally, a recently published study comprising obese individuals revealed that GLP-1 medications not only aid in weight loss but also reduce the risk of AD and other neurological disorders [13]. These studies provide valuable insights into the potential neuroprotective benefits of GLP-1 medications in the context of AD. However, much less is known about GLP-2 and its possible role in dementia and AD.

GLP-2, secreted mainly through intestinal L cells and preproglucagonergic (PPG) neurons in the brainstem and hypothalamus, is primarily known for regulating energy homeostasis by influencing intestinal motility and nutrient absorption [14]. Its role in the central nervous system (CNS) remains less explored; however, emerging evidence suggests that GLP-2 may act as a neuroprotective agent and play a key role in neurophysiology [15].

The GLP-2 receptor (GLP-2R) has been localized not only in the gut but also in the CNS, including the hypothalamus, hippocampus, and cortex. Initial in vitro studies suggest that GLP-2R activation protects neurons from cytotoxic damage [16], reduces cell death, and functions as a trophic factor for astrocytes by promoting their proliferation [17]. Notably, GLP-2R activation in neurons stimulates de novo protein synthesis and is involved in critical processes, including cell growth, proliferation, survival, angiogenesis, and protein production [17]. Therefore, GLP-2-induced protein synthesis may be crucial for maintaining neuronal survival and facilitating the action of secondary mediators, including growth factors and neuropeptides.

Moreover, studies using knockout rat models have shown that the absence of GLP-2 receptor stimulation is associated with spatial cognitive dysfunction following chronic cerebral hypoperfusion (CCH), while increased GLP-2R expression prevents CCH-induced cognitive impairment. Furthermore, enhanced GLP-2R expression has been linked to improved long-term memory and reduced depressive symptoms, both of which are commonly observed in AD [16].

Despite significant advances in understanding AD mechanisms, current diagnostic capabilities remain insufficient. While precise methods to diagnose exist, including neuropsychological tests (e.g., the Mini-Mental State Examination [MMSE] and the Addenbrooke’s Cognitive Examination), neuroimaging techniques (positron emission tomography [PET] and magnetic resonance imaging [MRI]), and cerebrospinal fluid (CSF) protein analysis, their application is often limited due to invasiveness, cost, and time constraints [18,19]. PET imaging is expensive and available only at specialized centers, making it unlikely to be widely adopted for routine assessment of cognitive impairment [19]. Although CSF sampling is becoming increasingly common in clinical practice, lumbar punctures remain a highly invasive procedure [20].

Over recent decades, numerous biomarkers have been identified in body fluids, but only a few plasma biomarkers have demonstrated significant diagnostic relevance for AD [21]. Consequently, there is a growing need for an effective, simple, and affordable AD screening tool suitable for outpatient settings. Blood biomarkers, in particular, offer a relatively non-invasive diagnostic approach with minimal associated risks.

To our knowledge, data on GLP-2 levels in humans with AD are currently lacking. Therefore, we aimed to determine plasma GLP-2 concentrations and assess whether GLP-2 could serve as a novel, potential, non-invasive biomarker for sporadic Alzheimer’s disease.

## 2. Results

### 2.1. Group Comparisons at Baseline

In the first phase of the study, significant differences were observed between patients with Alzheimer’s disease and cognitively healthy controls. Participants in the AD group were significantly older than those in the control group (79.13 ± 7.359 years vs. 63.61 ± 11.07 years; *p* < 0.0001 and had a shorter mean height (159.5 ± 9.157 cm vs. 164.2 ± 8.248 cm; *p* < 0.0355). Other anthropometric parameters, including weight, body mass index (BMI), and waist circumference did not differ significantly between the groups.

Regarding metabolic parameters, no significant differences were found between n the AD group and controls, except for high-density lipoprotein (HDL) cholesterol, which was significantly lower in the AD group (61.16 ± 13.95 mg/dL vs. 70.68 ± 17.44 mg/dL; *p* < 0.0485). Baseline plasma GLP-2 concentrations differ between the AD and control groups (2.767 ± 1.488 ng/mL vs. 1.941 ± 1.058 ng/mL; *p* = 0.0148).

Plasma GLP-2 levels differed modestly between AD patients (M = 2.77, SD = 1.49, n = 61) and controls (M = 1.94, SD = 1.06, n = 23). The effect size was moderate (Cohen’s d = 0.60), indicating a noticeable but not large difference between groups. A post hoc power analysis performed in G*Power (version 3.1.9.7) demonstrated that, given the observed effect size and group sizes, the achieved statistical power was approximately 68%, which is below the conventional 80% benchmark for adequate power. A sensitivity analysis further showed that an effect size of approximately d ≈ 0.63–0.67 would be required to reach 80% power with the present sample sizes. These results suggest that although a group difference is evident, the study had limited ability to reliably detect effects smaller than moderate magnitude, warranting cautious interpretation of the findings.

Detailed group characteristics are presented in Table 1.

### 2.2. Subgroup Analysis Based on Disease Severity

In the second phase of the analysis, the AD group was stratified into two subgroups according to disease severity: mild AD and moderate-to-severe AD. This stratification did not reveal significant differences in selected metabolic and anthropometric parameters within or between AD subgroups and controls.

Statistically significant differences in GLP-2 concentrations were observed in the AD subgroups when compared with the control group (for mild AD vs. controls 2.870 ± 1.554 ng/dL vs. 1.941 ± 1.058 ng/dL, *p* < 0.0250; and for moderate + severe AD vs. controls 2.628 ± 1.451 ng/dL vs. 1.941 ± 1.058 ng/dL, *p* < 0.0489, respectively).

All relevant data are summarized in Table 2.

### 2.3. Longitudinal Analysis of GLP-2 and Cognitive Function

No significant differences were observed in GLP-2 concentration between time points (0-AD I vs. 6-AD II, *p* = 0.7138; 0-AD I vs. 12-AD III, *p* = 0.7531; 6-AD II vs. 12-ADI vs. 12-AD III, *p* = 0.9976).

A one-way repeated-measures ANOVA was performed to evaluate plasma GLP-2 levels across three time points in AD patients (AD I, AD II, AD III; n = 34). Mean GLP-2 concentrations [ng/mL] were similar across measurements (2.960 ± 1.362, 3.101 ± 1.568, and 3.089 ± 1.747, respectively). The observed effect size was extremely small (f ≈ 0.022), indicating negligible temporal variation in GLP-2 levels. A post hoc power analysis conducted in G*Power 3.1.9.7 showed that, given the observed effect magnitude and sample size, the achieved statistical power was very low (≈5%). Sensitivity analysis further indicated that an effect size of approximately f ≈ 0.22 would be required to reach 80% power. These results demonstrate that the study was underpowered to detect very small changes in GLP-2 levels over time, and the absence of significant effects likely reflects both the minimal biological variation and insufficient statistical power.

**Figure 1:**
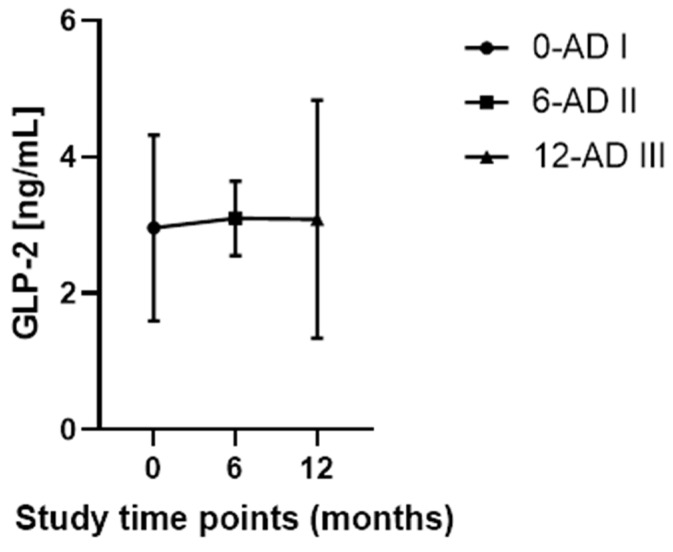
Longitudinal changes in plasma GLP-2 concentrations in patients with Alzheimer’s disease (AD). Plasma 2 levels were measured at baseline (0 months; AD I, n = 34), 6 months (AD II, n = 34), and 12 months (AD III, n = 34). Data are presented as mean ± standard deviation (SD). Statistical analysis was performed using one-way repeated measures ANOVA followed by Tukey’s post hoc test.

The data are illustrated in Figure 1.

Cognitive assessments indicated a progressive decline in patients with AD.

**Figure 2:**
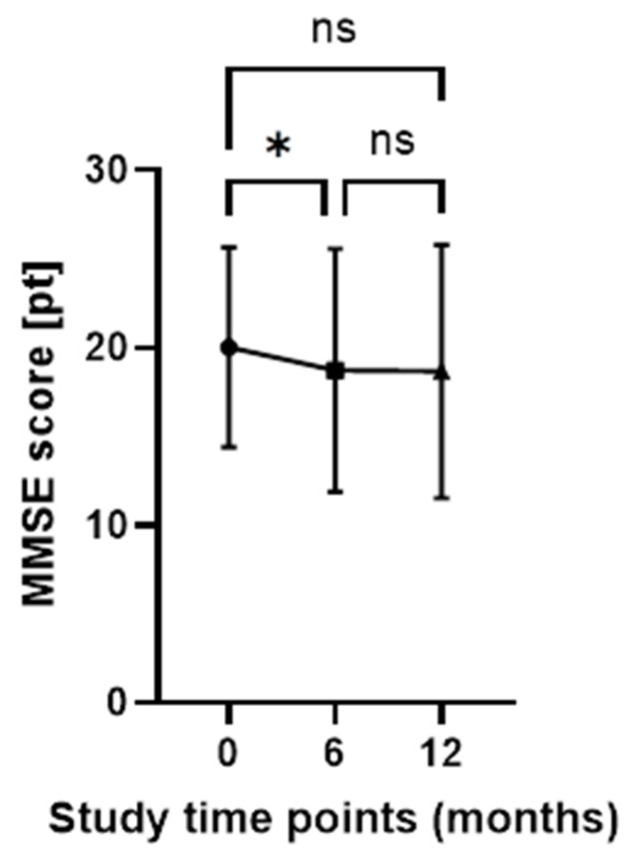
Mini-Mental State Examination (MMSE) scores over time in patients with Alzheimer’s disease (AD). MMSE scores were assessed at three time points: baseline (0 months; AD I, n = 34), 6 months (AD II, n = 34), and 12 months (AD III, n = 34). Data are presented as mean ± standard deviation (SD). Statistical analysis was performed using one-way repeated measures ANOVA followed by Tukey’s post hoc test. Ns—non-significant, * *p* = 0.0364.

The mean MMSE score differed significantly between time points 0 and 6 months (0-AD I vs. 6-AD II, *p* = 0.0364) while comparison of other time-point combinations showed no differences (0-AD I vs. 12-AD III, *p* = 0.0543; 6-AD II vs. 12-AD III, *p* = 0.9903, respectively). (Figure 2). The mean CDR score increased from 0.94 to 1.1 over the same period.

### 2.4. Correlation and Regression Analyses

Spearman correlation analyses revealed that plasma GLP-2 levels showed statistically significant positive associations with several metabolic parameters. Specifically, GLP-2 correlated with BMI (r = 0.3126, *p* = 0.0142), body weight (r = 0.3408, *p* = 0.0072), Homeostatic Model Assessment of Insulin Resistance (HOMA-IR) (r = 0.3394, *p* = 0.0074), and insulin levels (r = 0.3212, *p* = 0.0116). Table 3.

**Figure 3:**
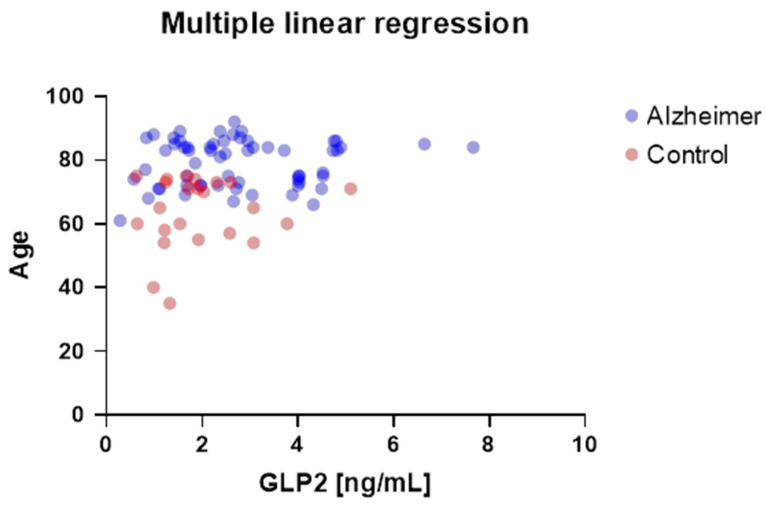
Scatterplot illustrating the relationship between age and plasma GLP-2 concentration in patients with Alzheimer’s disease (blue color, n = 61) and controls (red color, n = 23). Each dot represents an individual subject. The regression model included ‘group’ as a categorical variable and ‘age’ as a continuous covariate. Multiple linear regression analysis showed no significant effect of age or clinical group on GLP-2 levels (both *p* > 0.05).

A multiple linear regression model was applied to examine the influence of age and clinical group (AD vs. control) on GLP-2 levels. The analysis revealed that neither variable had a significant effect on GLP-2 concentration (both *p* > 0.05). These results are presented in Figure 3.

## 3. Discussion

Glucagon-like peptide-2 is primarily recognized for its role in promoting intestinal mucosal growth and enhancing nutrient absorption [22]. However, accumulating evidence suggests that GLP-2 may also exert significant neuroprotective effects. Several experimental studies have investigated its protective mechanisms in the context of neurodegenerative disorders [15,23]. To date, no published data address circulating GLP-2 concentrations in individuals with Alzheimer’s disease.

In the present study, plasma GLP-2 concentrations were significantly higher in patients with Alzheimer’s disease than in cognitively unimpaired controls (2.767 ± 1.488 vs. 1.941 ± 1.058 ng/mL; *p* = 0.0148). When stratified by disease severity, both mild and moderate-to-severe AD subgroups showed higher GLP-2 levels than controls (*p* = 0.0250 and *p* = 0.0489, respectively), with no significant difference between the two AD subgroups. The observed effect size was moderate (Cohen’s d ≈ 0.60), although the achieved post hoc power was approximately 68%, suggesting limited ability to detect effects smaller than moderate magnitude. Importantly, GLP-2 levels were positively associated with BMI, body weight, insulin concentrations, and HOMA-IR, indicating that circulating GLP-2 may be more closely linked to metabolic status than to clinical dementia severity.

The “type 3 diabetes” concept has been introduced to emphasize mechanistic convergence between type 2 diabetes mellitus (T2DM) and Alzheimer’s disease, in which brain insulin resistance and related metabolic stressors contribute to neurodegenerative signaling. Consistent with this framework, AD brains show impaired insulin/IGF-1 signaling accompanied by increased inhibitory IRS-1 (insulin receptor substrate 1) serine phosphorylation, changes reported even in the absence of overt diabetes and compatible with the notion of “brain insulin resistance” [24]. At the intracellular level, attenuated IRS-1/PI3K (phosphoinositide 3-kinase) signaling reduces Aktactivation, shifting the balance away from pro-survival and synaptotrophic programs [24,25]. Reduced Akt activity may, in turn, alter the inhibitory control of GSK-3β (glycogen synthase kinase 3 beta), thereby favoring tau phosphorylation and facilitating the emergence of neurofibrillary pathology, with secondary effects on microtubule stability, axonal transport, and synaptic integrity [24,25].

In parallel, insulin resistance may disrupt neuronal energy homeostasis by impairing GLUT-3/4 (glucose transporter type 3/4) trafficking and glucose utilization, thereby aggravating mitochondrial dysfunction and reactive oxygen species generation. These metabolic deficits can compromise ATP-dependent proteostasis and amyloid-β clearance, facilitating amyloid accumulation and synaptotoxicity. In addition, carbonyl stress and advanced glycation end-products (AGEs) may activate RAGE (receptor for advanced glycation end-products) and trigger NF-κB (nuclear factor kappa B)–dependent inflammatory signaling, reinforcing a feed-forward loop between oxidative stress and neuroinflammation. The AGE–RAGE axis also engages MAPK (mitogen-activated protein kinase) pathways (including ERK1/2 (extracellular signal-regulated kinase 1/2) and stress-activated signaling (JNK/STAT (c-Jun N-terminal kinase/signal transducer and activator of transcription), converging on transcriptional regulators such as NF-κB, AP-1 (activator protein 1), HIF-1α (hypoxia-inducible factor 1-alpha), and TGF-β (transforming growth factor beta). Through these mechanisms, inflammation can further impair IRS-1/PI3K/Akt signaling, creating a self-amplifying network linking insulin signaling failure with neuroinflammatory and amyloidogenic processes [26].

Collectively, this mechanistic architecture provides a biologically plausible route by which systemic and cerebral insulin resistance may lower the threshold for synaptic failure and accelerate neuronal loss in AD [24,25].

We failed to find any significant differences in insulin concentration between AD individuals and their non-demented counterparts. Based on the literature, plasma insulin findings in AD are contradictory, but several reports support a clinically relevant signal. In an ADNI-based study across the AD spectrum, higher plasma insulin levels were associated with worse cognitive performance and greater brain atrophy (particularly in AD-vulnerable regions), suggesting that hyperinsulinemia/IR may contribute to neurodegeneration [27]. Overall, available evidence supports the view that insulin abnormalities can be present in AD. Still, the direction and magnitude of peripheral changes in circulating insulin likely depend on disease stage, comorbidities (T2D/obesity), and other factors [28]. Genetic evidence may further support a role for metabolic factors: in a 2024 Mendelian randomization analysis, genetically predicted fasting insulin (but not diabetes status per se) was associated with AD dementia risk [29]. Beyond insulin levels alone, higher peripheral IR (e.g., HOMA-IR) has been associated with lower regional cerebral glucose metabolism on FDG-PET in late middle-aged adults at risk for AD, aligning systemic metabolic dysfunction with AD-vulnerable brain hypometabolism [30].

It should be noted that there is also a mechanistic bridge between the IR and incretin pathways in AD. In detail, GLP-1 receptor agonists improve systemic insulin sensitivity and have direct CNS effects in experimental models (affecting anti-inflammatory signaling, mitochondrial and synaptic support, and potentially impacting protein homeostasis) [25].

GLP-2 was not correlated with MMSE or CDR scores. Furthermore, in multivariable regression models that included age and clinical group, neither variable independently predicted GLP-2 concentrations (both *p* > 0.05), highlighting the potential influence of metabolic and other unmeasured factors. Overall, elevated GLP-2 levels in AD may reflect a compensatory or stress-related response; however, the clinical significance of this finding requires confirmation in larger and metabolically phenotyped cohorts.

Longitudinal assessments of cognitive performance, using the Mini-Mental State Examination and the Clinical Dementia Rating scale, confirmed disease progression across the study period. Importantly, no significant associations were observed between plasma GLP-2 levels and MMSE or CDR scores, suggesting that changes in GLP-2 concentrations were not associated with cognitive decline in our cohort.

The relatively small sample size, particularly at the study’s conclusion, represents a potential limitation that may have impacted the statistical power of our analyses. Nevertheless, given the exploratory nature of this investigation, these findings should be interpreted as preliminary. Future studies involving larger and more diverse populations are warranted. In addition, subsequent research should include individuals with mild cognitive impairment (MCI), an intermediate clinical stage between age-related cognitive changes and overt dementia, particularly AD. MCI is characterized by deficits in one or more cognitive domains, including but not limited to memory [31]. Approximately 50% of individuals with MCI progress to AD within five years, although MCI may also represent a prodromal phase of other dementias [32]. Therefore, we plan to continue the study with a larger cohort of patients with Alzheimer’s disease, as well as to expand it to include individuals with mild cognitive impairment.

Although clinical data regarding the role of GLP-2 in dementia, including AD, remain scarce, preclinical evidence suggests that GLP-2 and its analogues may confer neuroprotection through multiple mechanisms. These mechanisms encompass modulation of neurogenesis, oxidative stress, inflammation, mitochondrial function, and synaptic plasticity [33,34,35]. Experimental studies have demonstrated that GLP-2 receptors (GLP-2R) are expressed in several brain regions, albeit with a more restricted and less well-characterized distribution compared to GLP-1 receptors. GLP-2R has been identified in the hippocampus (CA1 (Cornu Ammonis 1), CA3 Cornu Ammonis 1), dentate gyrus), cerebral cortex, and brainstem (nucleus of the solitary tract) [34,36,37]. Notably, the hippocampus—particularly regions implicated in memory consolidation, learning, and adult neurogenesis—appears to be a significant site of GLP-2 activity. GLP-2 may facilitate neural progenitor proliferation (↑ Ki67, DCX (doublecortin)), upregulate neurotrophic factors such as BDNF (brain-derived neurotrophic factor), and support long-term potentiation (LTP) and long-term depression (LTD) [33]. GLP-2R expression has also been detected in neurons, astrocytes, microglia, and neural progenitor cells, suggesting a potential role in modulating inflammatory activation and promoting neurogenesis [15]. While GLP-2 demonstrates limited penetration across the blood–brain barrier (BBB), it may exert central effects indirectly via peripheral pathways, including vagal afferents and gut–brain axis signaling, particularly through the nucleus of the solitary tract [38].

Multiple mechanisms underlying the neuroprotective actions of GLP-2 have been proposed. GLP-2 appears to attenuate microglial and astrocytic overactivation, thereby reducing the release of pro-inflammatory cytokines, including IL-1β, TNF-α, and IL-6 [34,39]. Furthermore, GLP-2 enhances antioxidant defenses by upregulating enzymes such as superoxide dismutase (SOD) and catalase, thereby decreasing the accumulation of reactive oxygen species and mitigating oxidative damage [15,34,39]. In addition, GLP-2 has been shown to preserve mitochondrial function, counteracting mitochondrial dysfunction, autophagy impairments, and apoptotic processes, thereby preventing neuronal energy failure and cell death [35]. Experimental evidence further indicates that GLP-2 reduces neuronal apoptosis in both in vitro and in vivo models, suppressing caspase-3 activation and cytochrome c release while enhancing anti-apoptotic pathways [40]. GLP-2-mediated neuroprotection also involves the activation of signaling pathways critical for neuronal survival, including the PI3K/Akt and MAPK/ERK pathways, which are implicated in synaptic plasticity and memory processes [16,41,42]. Indeed, GLP-2R activation in the CNS stimulates the PI3K/Akt/mTOR pathway, facilitating protein synthesis, dendritic growth, synaptogenesis, and neurogenesis [43]. Upregulation of neurogenesis markers, such as Ki-67, DCX, and NeuN (neuronal nuclei), further supports this mechanism [33].

**Figure 4:**
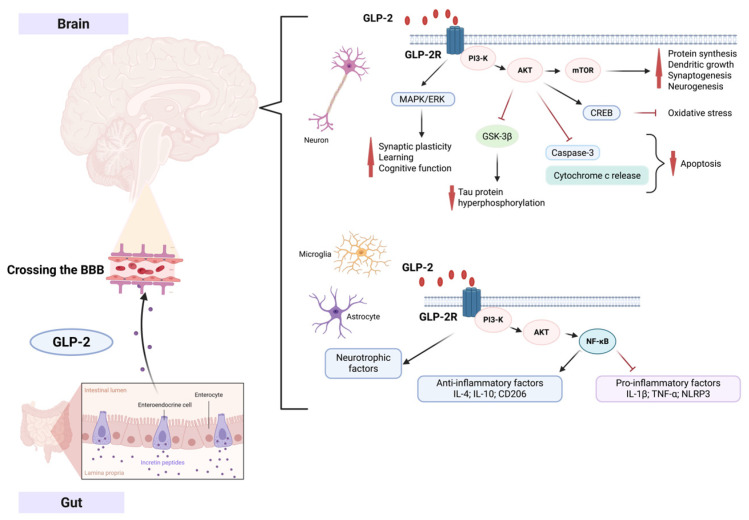
Scheme of the potential neuroprotective action of GLP-2. GLP-2 (glucagon-like peptide-2), produced in the gut, crosses the blood–brain barrier (BBB) and acts via GLP-2 receptors (GLP-2R) on neurons, astrocytes, and microglia. In neurons, GLP-2R activation enhances synaptic plasticity, cognitive function, and neuronal survival through MAPK/ERK, PI3-K/AKT, and mTOR signaling, while reducing tau hyperphosphorylation and apoptosis. In glial cells, GLP-2 modulates neuroinflammation by increasing anti-inflammatory (IL-4, IL-10) and neurotrophic factors, and suppressing pro-inflammatory cytokines (IL-1β, TNF-α, NLRP3). Created in BioRender. Litwiniuk, A. (2026) https://BioRender.com/vu1p6ft (accessed on 8 January 2026). Abbreviations: GLP-2–glucagon-like peptide-2; GLP-2R–GLP-2 receptor; BBB–blood–brain barrier; PI3-K–phosphoinositide 3-kinase; AKT–protein kinase B; MAPK/ERK–mitogen-activated protein kinase/extracellular signal-regulated kinase; mTOR–mammalian target of rapamycin; IL–interleukin; TNF-α–tumor necrosis factor-alpha; NLRP3–NOD-, LRR- and pyrin domain-containing protein 3.

A summary of the potential neuroprotective effects of GLP-2 is presented in Figure 4.

Glucagon-like peptide-2 has been shown to have neuroprotective effects in several preclinical models of neurodegeneration. In rodents fed a high-fat diet, GLP-2 administration significantly reduced glial fibrillary acidic protein (GFAP) expression, a marker of reactive gliosis, and attenuated neurodegenerative changes (including reductions in NF-κB, pro-inflammatory cytokines, amyloid precursor protein, oxidative stress markers, and neuronal apoptosis) [34]. Additionally, GLP-2 has been shown to support blood–brain barrier integrity, partly of vascular endothelial growth factor (VEGF), which promotes angiogenesis and stabilizes the BBB [44,45].

Experimental studies using animal models of neurodegenerative diseases further corroborate the neuroprotective potential of GLP-2 analogues. In a murine model of sporadic Alzheimer’s disease induced by intracerebroventricular administration of streptozotocin, GLP-2 improved spatial learning in the Morris Water Maze test, normalized oxidative stress parameters (elevated glutathione, reduced thiobarbituric acid reactive substances), and promoted hippocampal neurogenesis, as evidenced by increased expression of Ki67 and doublecortin [33]. In a model of vascular dementia caused by chronic cerebral hypoperfusion, treatment with teduglutide—a dipeptidyl peptidase-4 (DPP-4)-resistant GLP-2 analogue—restored hippocampal GLP-2 receptor expression, improved long-term potentiation and long-term depression, and ameliorated cognitive performance [16]. In another study, long-term administration of Gly^2^-GLP-2 to mice on a high-fat diet attenuated oxidative stress, reduced markers of neuroinflammation (GFAP (glial fibrillary acidic protein), nuclear factor kappa B, amyloid precursor protein), and improved hippocampal function and cognition [34].

Further evidence comes from models of Parkinson’s disease (PD). In the MPTP (1-methyl-4-phenyl-1,2,3,6-tetrahydropyridine)-induced mouse model, Gly^2^-GLP-2 improved motor coordination, preserved mitochondrial function, reduced neuroinflammatory activity, and inhibited NLRP3 (NOD-, LRR- and pyrin domain-containing protein 3) inflammasome activation [23]. In vitro studies using SH-SY5Y and Neuro-2a neuronal cell lines exposed to mitochondrial toxins demonstrated that GLP-2 preserved mitochondrial membrane potential and ATP production, reduced apoptotic signaling (via caspase-3 suppression and Bcl-2 upregulation), and enhanced autophagic flux [35].

Despite these promising findings, clinical translation of GLP-2-based therapies remains limited. Currently, GLP-2 analogues are approved only for short bowel syndrome [46], and no clinical trials have evaluated their efficacy in neurodegenerative conditions. Challenges to clinical application include limited BBB penetration and a lack of safety and efficacy data in neurological populations. These factors may limit the translational potential of GLP-1 receptor agonists.

In contrast, GLP-1 and its receptor agonists have been extensively investigated for neuroprotective effects, particularly in Alzheimer’s disease. GLP-1 is produced not only in the gut but also in the brainstem (nucleus tractus solitarius), and its receptors are widely distributed in the hippocampus and cortex [47,48]. Activation of GLP-1 receptors counteracts multiple pathogenic mechanisms implicated in AD, including insulin resistance, mitochondrial dysfunction, oxidative stress, neuroinflammation, and neuronal apoptosis [49,50].

Preclinical studies using APP/PS1 (amyloid precursor protein/presenilin-1) and triple transgenic (3xTg)-AD mouse models have consistently demonstrated that GLP-1 RAs, such as liraglutide, semaglutide, and exendin-4, reduce β-amyloid accumulation, inhibit tau phosphorylation, restore synaptic plasticity, enhance neurogenesis, and improve cognitive outcomes [51,52,53]. These effects are independent of glycemic control, indicating a direct central mechanism of action. GLP-1 also restores insulin signaling via the IRS-1/PI3K/Akt pathways, enhances mitochondrial function by reducing reactive oxygen species production, and suppresses neuroinflammation by downregulating pro-inflammatory cytokines and microglial activity [50].

Clinical data, though still emerging, are encouraging. Currently, clinical trials are being conducted in individuals with Alzheimer’s disease to investigate the effects of various GLP-1 analogues on cognitive function, cerebral glucose metabolism, disease biomarkers, and treatment tolerance and safety [54]. Additionally, some observational studies on GLP-1 analogues have assessed their impact on cognitive deficits. However, observational findings are scarce due to the lack of granular information about cognitive scores in routinely collected data [54].

Below, we present the available data on studies investigating the roles of GLP-1 and GLP-2 in Alzheimer’s disease (Table 4).

Taken together, while both GLP-1 and GLP-2 share several mechanistic targets—such as modulation of neuroinflammation, oxidative stress, and mitochondrial protection—GLP-1 RAs benefit from superior blood–brain barrier penetration, broader receptor distribution in the CNS, and a more advanced stage of clinical development. Their potential as disease-modifying therapies is currently under investigation in large-scale randomized trials. Comparative analyses of GLP-1 and GLP-2 pathways may yield insights into complementary or synergistic therapeutic strategies for neurodegenerative diseases.

## 4. Materials and Methods 

### 4.1. Material

The study consisted of two phases. In the first phase, a cross-sectional comparison was conducted between patients diagnosed with Alzheimer’s disease and cognitively healthy controls. The second phase consisted of a longitudinal observational analysis aimed at assessing potential changes in plasma GLP-2 concentrations over a 12-month follow-up period in individuals with Alzheimer’s disease.

For this study, we initially randomly enrolled 61 patients (39 women and 22 men) who were diagnosed with Alzheimer’s disease at the Neuroprotect Medical Center in Warsaw, Poland. The inclusion and exclusion criteria detailed below were applied. The same group of AD individuals participated in both arms of the study. For the first arm of the study, a control group of 23 individuals (17 women and 6 men) without neurodegenerative conditions affecting cognitive function was included. Cognitive function was normal, as confirmed by the MMSE.

The average age of the patients was 80 years (79.13 ± 7.36 years). The control group consisted of 23 individuals who had been excluded from having dementia (aged 63.61 ± 11.07 years). 

### 4.2. Study Design

The study was conducted in the following main stages:

1. Recruitment of Patients Based on Established Criteria:

Patients diagnosed with AD were categorized into three subgroups based on disease severity using the Mini-Mental State Examination (MMSE) and the Clinical Dementia Rating (CDR) scales, following guidelines from the National Institute on Aging (NIA) and the Alzheimer’s Association (AA).

Inclusion Criteria:

  ○ Diagnosis of AD-related dementia at mild, moderate, or severe stages:
    ▪ Mild AD: MMSE 19–26, CDR 0.5 or 1
    ▪ Moderate AD: MMSE 11–18, CDR 1 or 2
    ▪ Severe AD: MMSE < 10, CDR 2 or 3
  ○ Age > 60 years at the initial visit
  ○ Written informed consent obtained from the patient and/or their legal representative
  ○ Presence of at least one adult caregiver capable of providing reliable assessments and complying with study procedures
  ○ At least 60 days before the study:
    ▪ No use of pro-cognitive drugs such as acetylcholinesterase inhibitors (AChEI) or memantine, or
    ▪ Treatment with either AChEI or memantine at a stable dose throughout the study period

Exclusion Criteria:

  ○ Presence of significant CNS disorders other than AD (e.g., Lewy body dementia, Parkinson’s disease (PD), multiple sclerosis, Huntington’s disease, traumatic brain injury, or brain tumors)
  ○ Vascular dementia
  ○ Prior epilepsy diagnosis
  ○ History of major depressive disorder, psychotic disorders, or bipolar disorder within the past five years
  ○ Lifetime diagnosis of schizophrenia
  ○ Substance or alcohol dependency within the past two years
  ○ Residence in a moderate- or high-dependency care facility
  ○ Significant systemic conditions deemed contraindications by the investigator
  ○ Vitamin B12 and/or folate deficiency affecting cognitive function
  ○ Abnormal laboratory results precluding participation
  ○ Malignant neoplasm diagnosed within the past five years (exceptions: fully treated basal cell carcinoma, squamous cell carcinoma, or stage I prostate cancer)
  ○ Treatment with drugs affecting cognitive function within 48 h before psychological testing
  ○ Treatment with GLP-1 analogues or tirzepatide (dual GLP-1/GIP analogues) within 3 months before blood tests and study period
  ○ Active hepatitis B/C or positive HIV test
  ○ Chronic systemic steroid or immunosuppressive treatment
  ○ Blood donation within eight weeks before and during the study

2. Study Follow-up (for the second arm of the study):

Three visits were scheduled every six months—AD I (0) as the first visit, AD II (6 months) as the second visit, and AD III (12 months) as the third visit. During these visits, blood samples were collected—initially, a comprehensive panel was taken, and GLP-2 assessments were conducted at each visit. Patients were also evaluated using the MMSE and CDR scales. Psychological assessments were performed at the Neuroprotect Medical Center by trained psychologists. For the longitudinal analysis of GLP-2 and MMSE, complete data were available for 34 participants at each time point (baseline n = 34; 6 months n = 34; 12 months n = 34). The sex distribution included 22 women and 12 men.

3. Blood Sample Collection

Blood samples were collected between 8:00 a.m. and 9:00 a.m. following a 12-h fast. Anthropometric measurements (weight, height, BMI) were also recorded.

  ○ Venous blood was collected into tubes containing EDTA and aprotinin (protease inhibitor) (Becton Dickinson, Franklin Lakes, NJ, USA). The samples were then centrifuged at 4 °C, and the collected plasma was used for measuring GLP-2 levels. Immediately, after centrifugation (4 °C, 3000 rpm for 10 min), plasma aliquots (0.5 mL) were stored at −80 °C until analysis.
  ○ Venous blood was also collected for routine blood parameter assessment (initial testing included hematology, liver function tests, creatinine, lipid panel, glucose, and TSH). Non-frozen samples were quickly sent to the laboratory for further analysis.

4. GLP-2 Plasma Level Assessment

The concentration of total GLP-2 in plasma was determined in non-diluted samples using commercial enzyme-linked immunosorbent assay (ELISA) kits (Cat. No. EZGLP2-37K; EMD Millipore Corporation, Danvers, MA, USA). This assay quantifies total GLP-2, encompassing both the intact (active) GLP-2 (1–33) and the DPP-4–cleaved inactive form of GLP-2 (3–33). The sensitivity of the GLP-2 assay was 0.3 ng/mL. The intra-assay and inter-assay coefficients of variation (CV) were 3.0–9.1% and 3.3–11.5%, respectively.

5. Insulin Plasma Level Assessment

The concentration of insulin in plasma was determined in non-diluted samples using commercial ELISA kits (Cat. No. EIA-2935; DRG Instruments GmbH, Marburg, Germany). The sensitivity of the insulin assay was 1.76 µIU/mL. The intra-assay and inter-assay coefficients of variation (CV) were 1.8–2.6% and 2.9–6.0%, respectively.

6. Routine Tests

Hematology, creatinine, liver function, lipid panel, glucose, and TSH were assessed using standard laboratory methods.

7. HOMA-IR (Homeostatic Model Assessment of Insulin Resistance)

HOMA-IR was calculated according to the formula: (Fasting Glucose (mg/dL) × Fasting Insulin (µUm/L))/405.

### 4.3. Statistical Analysis

Statistical analyses were performed using GraphPad Prism 10.6.1. The normality of data distribution was assessed using the Shapiro–Wilk test. Depending on data distribution, appropriate parametric or non-parametric tests were used, including Student’s *t*-test, Mann–Whitney U test, Kruskal–Wallis test, and Spearman correlation. A *p*-value of <0.05 was considered statistically significant.

Continuous variables are presented as mean ± standard deviation (SD). MMSE scores and plasma GLP-2 concentrations in patients with Alzheimer’s disease, measured at 6-month intervals, were analyzed using a one-way repeated-measures ANOVA followed by Tukey’s post hoc test. Multiple linear regression was used to assess the effect of age and clinical group (Alzheimer’s vs. control) on plasma GLP-2 levels. Statistical significance was defined as *p* < 0.05.

The effect size for group comparisons was quantified using Cohen’s d, calculated as the difference between group means divided by the pooled standard deviation. Post hoc statistical power was estimated in G*Power (version 3.1.9.7) using a two-tailed independent-samples *t*-test, based on the observed effect size, α = 0.05, and the actual sample sizes. These procedures were applied to assess the magnitude of group differences and the achieved power of the analysis.

### 4.4. Ethical Considerations

The study was approved by the Bioethics Committee of the Centre of Postgraduate Medical Education, Warsaw, Poland (31/PB/2020) on 11 March 2020. All participants or their legal representatives provided informed consent before participation. The study was conducted in accordance with the principles outlined in the Declaration of Helsinki.

## 5. Conclusions

Despite promising preclinical data, clinical translation of GLP-2-based interventions remains limited. At present, GLP-2 analogues are approved for the treatment of short bowel syndrome, and no clinical trials have evaluated GLP-2-related therapies in neurodegenerative disorders. Key barriers include limited central nervous system exposure due to peptide-related constraints and insufficient safety and efficacy data in neurological populations. These factors may pose greater translational challenges than those posed by incretin-based therapies such as GLP-1 receptor agonists, for which clinical development in neurological indications is more advanced.

The present study is among the first to assess circulating GLP-2 concentrations in patients with Alzheimer’s disease. Plasma GLP-2 levels were higher in Alzheimer’s disease than in cognitively unimpaired controls; however, GLP-2 was not associated with cognitive performance or its change over 12 months. In contrast, GLP-2 showed positive associations with markers of adiposity and insulin resistance, suggesting that circulating GLP-2 may be more strongly linked to metabolic status than to clinical dementia severity. Future studies should replicate these findings in larger and age-matched cohorts with detailed metabolic phenotyping, evaluate longitudinal changes with adequate statistical power, and determine whether GLP-2 provides independent diagnostic or prognostic value beyond metabolic covariates.

## Figures and Tables

**Table 1:** Baseline characteristics of the studied groups, including anthropometric measurements, biochemical markers, and GLP-2 concentrations assessed in the blood of patients with AD and control participants. Data are presented as mean ± standard deviation (SD). Differences between groups were analyzed using Student’s *t*-test for parametric data or the Mann–Whitney *U* test for non-parametric data, as appropriate. A *p*-value < 0.05 was considered statistically significant.

	Patients with AD (n = 61)	Controls (n = 23)	*p*
AGE [YEARS]	79.13 ± 7.359	63.61 ± 11.07	*p* < 0.0001
HEIGHT [CM]	159.5 ± 9.157	164.2 ± 8.248	*p* = 0.0355
WEIGHT [KG]	66.78 ± 14.61	73.42 ± 10.91	*p* = 0.0515
BMI (BODY MASS INDEX) [KG/M^2^]	26.05 ± 4.791	27.22 ± 3.355	*p* = 0.2294
WAIST CIRCUMFERENCE [CM]	92.84 ± 12.34	93.83 ± 10.46	*p* = 0.8245
TOTAL CHOLESTEROL [MG/DL]	194.2 ± 54.18	209.8 ± 46.71	*p* = 0.1814
HDL (HIGH-DENSITY LIPOPROTEIN) [MG/DL]	61.16 ± 13.95	70.68 ± 17.44	*p* = 0.0485
LDL (LOW-DENSITY LIPOPROTEIN) [MG/DL]	111.6 ± 46.78	118.00 ± 41.53	*p* = 0.5044
TRIGLYCERIDES [MG/DL]	107.3 ± 39.80	106.9 ± 46.24	*p* = 0.8273
GLUCOSE [MG/DL]	105.5± 31.15	93.13 ± 7.568	*p* = 0.2069
INSULIN [µIU/ML]	10.27 ± 9.744	12.24 ± 7.065	*p* = 0.0568
HOMA-IR	3.122 ± 4.709	2.850 ±1.699	*p* = 0.1391
GLP-2 [NG/ML]	2.767 ± 1.488	1.941± 1.058	*p* = 0.0148

**Table 2:** Baseline characteristics of the studied groups, including anthropometric measurements, biochemical markers, and GLP-2 concentrations in blood samples from subgroups of patients with AD and control participants. Data are presented as mean ± standard deviation (SD). Statistical differences between subgroups were assessed using the Kruskal–Wallis test, followed, where appropriate, by the Mann–Whitney U test for pairwise comparisons. A *p*-value < 0.05 was considered statistically significant.

	I MILD AD (n = 33)	II MODERATE + SEVERE AD (n = 28)	III CONTROLS (n = 23)	*p*
BMI (BODY MASS INDEX) [KG/M^2^]	25.39 ± 3.925	27.25 ± 5.027	27.22 ± 3.355	I vs. III *p* = 0.1491 II vs. III *p* = 0.6557 I vs. II *p* = 0.3107
WAIST CIRCUMFERENCE [CM]	90.97 ± 13.05	95.05 ± 11.27	93.83 ± 10.46	I vs. III *p* = 0.5108 II vs. III *p* = 0.7459 I vs. II *p* = 0.3218
TOTAL CHOLESTEROL [MG/DL]	186.4 ± 47.24	203.4 ± 60.96	209.8 ± 46.71	I vs III *p* = 0.0729 II vs. III *p* = 0.6237 I vs. II *p* = 0.3117
HDL (HIGH-DENSITY LIPOPROTEIN) [MG/DL]	61.21 ± 15.56	61.11 ± 12.07	70.68 ± 17.44	I vs. III *p* = 0.0673 II vs. III *p* = 0.0990 I vs. II *p* = 0.8322
LDL (LOW-DENSITY LIPOPROTEIN) [MG/DL]	104.1 ± 40.45	120.4 ± 52.68	118.00 ± 41.53	I vs. III *p* = 0.2212 II vs. III *p* = 0.9084 I vs. II *p* = 0.3047
TRIGLYCERIDES [MG/DL]	105.5 ± 41.95	109.5 ± 37.76	106.9 ± 46.24	I vs. III *p* = 0.9424 II vs. III *p* = 06212 I vs. II *p* = 0.6200
GLUCOSE [MG/DL]	110.2 ± 35.71	99.83 ± 24.18	93.13 ± 7.568	I vs. III *p* = 0.1380 II vs. III *p* = 0.4899 I vs. II *p* = 0.3151
INSULIN [µIU/ML]	11.39 ± 12.16	9.084 ± 5.978	12.24 ± 7.065	I vs. III *p* = 0.0935 II vs. III *p* = 0.1091 I vs. II *p* = 0.7221
HOMA-IR	3.712 ± 6.021	2.428 ± 2.322	2.850 ±1.699	I vs. III *p* = 0.2059 II vs. III *p* = 0.1790 I vs. II *p* = 0.6023
GLP-2 [NG/ML]	2.870 ± 1.554	2.628 ± 1.451	1.941 ± 1.058	I vs. III *p* = 0.0250 II vs. III *p* = 0.0489 I vs. II *p* = 0.3626

**Table 3:** Spearman correlation analysis between GLP-2 levels and other parameters (cognitive assessment, metabolic results, and anthropometric measurements).

	GLP-2	
Variable	R Spearman Correlation	*p*
MMSE	0.1261	0.3328
CDR	−0.1125	0.4136
Insulin	0.3212	0.0116
Glucose	0.1824	0.1595
HOMA-IR	0.3394	0.0074
TOTAL CHOL	−0.1389	0.2857
HDL	−0.1786	0.1684
LDL	−0.1275	0.3273
Triglycerides	0.08158	0.5320
BMI	0.3126	0.0142
WEIGHT	0.3408	0.0072
HEIGHT	0.1232	0.3443
AGE	0.02962	0.8208
WAIST CIRCUMFERENCE	0.2331	0.0706

**Table 4:** GLP-1/GLP-2-related evidence in Alzheimer’s disease.

Category	Agent/Intervention	Study Design & Population/Model	AD/Dementia-Relevant Outcomes	Main Finding (Brief, Neutral)	Ref.
Human—interventional	Liraglutide (GLP-1RA)	Phase 2b, double-blind randomized trial (~12 months); mild–moderate AD	Neuroimaging/atrophy; biomarkers; cognition	Favorable signals in some neuroimaging endpoints; clinical outcomes require further confirmation.	[55]
Human—interventional	Liraglutide (GLP-1RA)	Randomized, placebo-controlled trial (26 weeks); mild AD	FDG-PET; cerebral glucose metabolism; cognition	Signal of slower decline in cerebral glucose metabolism; no clear clinical benefit over short follow-up.	[56]
Human—interventional	Exenatide (GLP-1RA)	Phase II, double-blind randomized placebo-controlled trial (18 months); early AD	Safety/tolerability; cognition/clinical outcomes; MRI and exploratory biomarkers	No clear differences vs. placebo on clinical/cognitive endpoints; exploratory biomarker signals were inconclusive.	[57]
Human—interventional	Oral semaglutide (GLP-1RA)	Phase 3 trial design report (EVOKE/EVOKE+); AD	Planned clinical endpoints: disease progression	Phase 3 trials were designed to test the slowing of clinical progression; topline results reported not meeting primary endpoints; full peer-reviewed data awaited.	[58]
Human—observational	Semaglutide (GLP-1RA)	Propensity-score matched cohort; adults with T2D (12-month follow-up)	Neurological and psychiatric outcomes (not dementia-specific)	Assessed 12-month neurological/psychiatric outcomes during semaglutide use; not specific to AD incidence.	[59]
Human—observational	Semaglutide (GLP-1RA)	Target-trial emulation (real-world data); adults with T2D	Incident AD diagnosis (target-trial emulation)	Associated with a lower risk of first-time AD diagnosis vs. selected therapies; observational design may be prone to bias.	[12]
Human—observational	GLP-1RAs vs SGLT2 inhibitors	Real-world comparative cohort: adults with T2D	Incident dementia/AD outcomes (comparative cohort)	Possible benefit in some comparisons; estimates were sensitive to comparator choice and confounding control.	[60]
Human—observational	GLP-1RAs vs other glucose-lowering drugs	Nationwide cohort/comparative effectiveness; adults with T2D	Incident dementia/AD outcomes (comparative effectiveness)	Dementia risk estimates varied by comparator class; conclusions limited by observational design.	[61]
Human—observational	Exenatide (GLP-1RA)	Retrospective cohort (claims/EHR); adults with T2D	Incident AD diagnosis (real-world data)	Associated with lower AD risk vs. other therapies; residual confounding remains possible.	[62]
Human—biomarkers	Endogenous GLP-1	Cross-sectional analysis: AD vs. controls	Circulating GLP-1; cognition; AD biomarkers	Circulating GLP-1 correlated with cognition and AD biomarkers; cross-sectional data do not support causal inference.	[63]
Preclinical	Liraglutide (GLP-1RA)	Transgenic AD mouse model	Neurodegeneration markers; pathology; behavior	Reduced neurodegeneration-related markers in transgenic AD models.	[64]
Preclinical	Liraglutide (GLP-1RA)	Aged APP/PS1 mouse model	Synapses; amyloid burden; behavior	Attenuated synapse loss and (in some models) amyloid plaque burden.	[65]
Preclinical	Semaglutide (GLP-1RA)/Tirzepatide (dual agonist GLP-1RA/GIP RA)	5×FAD and APP/PS1 mice	Pathology/behavior endpoints in AD models	No consistent improvement in neurodegeneration endpoints despite metabolic effects (model- and endpoint-dependent).	[66]
Preclinical	Liraglutide (GLP-1RA)	Long-term treatment in transgenic APP/PS1 mice	Aβ plaque burden; pathology	Reduced Aβ plaque load in transgenic APP/PS1 models following long-term treatment.	[67]
Preclinical	Exenatide (GLP-1RA)	5×FAD model	Mitochondrial function; synapses; behavior	Improved mitochondrial function and behavioral performance in a 5×FAD model.	[68]
Preclinical	NLY01 (PEGylated exenatide)	AD mouse models; glia-focused mechanistic study	Glial activation; neuroprotection; pathology	Modulated microglia–astrocyte crosstalk with neuroprotective effects in AD models.	[69]
Preclinical	Liraglutide (GLP-1RA)	Translational mouse + non-human primate work	Insulin/synaptic receptor markers (translational)	Favorable changes in insulin- and synapse-related markers in translational mouse and non-human primate work.	[52]
Preclinical	Liraglutide (GLP-1RA)	Combined AD + T2D murine model	Vascular/neuronal outcomes; cognition	Improved vascular/neuronal pathology and cognitive performance in combined AD + T2D models.	[70]
Preclinical	Semaglutide (GLP-1RA)	APP/PS1 mouse model	Inflammation; Aβ/tau-related pathways; behavior	Anti-inflammatory effects; mechanisms included AMPK activation and inhibition of TLR4/NF-κB signaling.	[71]
Preclinical	GLP-2	ICV-STZ dementia model (mice)	Cognition; oxidative stress; neurogenesis	Improved working memory, oxidative stress markers, and neurogenesis in an ICV-STZ model.	[33]
Preclinical	GLP2R	Chronic cerebral hypoperfusion model (rats)	Cognition; AKT–mTOR–p70S6K signaling	Attenuated cognitive deficits and modulated AKT–mTOR–p70S6K signaling in a chronic cerebral hypoperfusion model.	[16]

## Data Availability

The datasets presented in this article are not readily available because the data are part of an ongoing study. Requests to access the datasets should be directed to the corresponding author.

## Abbreviations

5×FAD	five familial Alzheimer’s disease mutations (5xFAD transgenic model)
Aβ	β-amyloid
AD	Alzheimer’s disease
ADNI	Alzheimer’s Disease Neuroimaging Initiative
AChEI	acetylcholinesterase inhibitor
AGE(s)	advanced glycation end-product(s)
AKT (Akt)	protein kinase B
AMPK	AMP-activated protein kinase
AP-1	activator protein 1
APP	amyloid precursor protein
APP/PS1	amyloid precursor protein/presenilin-1 transgenic model
ATP	adenosine triphosphate
BBB	blood–brain barrier
BDNF	brain-derived neurotrophic factor
BMI	body mass index
CA1	Cornu Ammonis 1 (hippocampal region)
CA3	Cornu Ammonis 3 (hippocampal region)
CCH	chronic cerebral hypoperfusion
CDR	Clinical Dementia Rating
CNS	central nervous system
CSF	cerebrospinal fluid
DCX	doublecortin
DPP-4	dipeptidyl peptidase-4
EDTA	ethylenediaminetetraacetic acid
ELISA	enzyme-linked immunosorbent assay
ERK	extracellular signal-regulated kinase
ERK1/2	extracellular signal-regulated kinase 1/2
FDG-PET	18F-fluorodeoxyglucose positron emission tomography
GFAP	glial fibrillary acidic protein
GIP	glucose-dependent insulinotropic polypeptide
GLP-1	glucagon-like peptide-1
GLP-1 RA/GLP-1RA	GLP-1 receptor agonist
GLP-2	glucagon-like peptide-2
GLP-2R	GLP-2 receptor
GLUT-3/4	glucose transporter type 3/4
GSK-3β	glycogen synthase kinase 3 beta
HBV	hepatitis B virus
HCV	hepatitis C virus
HDL	high-density lipoprotein
HIF-1α	hypoxia-inducible factor 1-alpha
HIV	human immunodeficiency virus
HOMA-IR	Homeostatic Model Assessment of Insulin Resistance
ICV	intracerebroventricular
ICV-STZ	intracerebroventricular streptozotocin model
IGF-1	insulin-like growth factor 1
IL	interleukin
IR	insulin resistance
IRS-1	insulin receptor substrate 1
JNK	c-Jun N-terminal kinase
JNK/STAT	c-Jun N-terminal kinase/signal transducer and activator of transcription
LDL	low-density lipoprotein
LPS	lipopolysaccharide
LTD	long-term depression
LTP	long-term potentiation
MAPK	mitogen-activated protein kinase
MCI	mild cognitive impairment
MMSE	Mini-Mental State Examination
MRI	magnetic resonance imaging
MPTP	1-methyl-4-phenyl-1,2,3,6-tetrahydropyridine
mTOR	mammalian target of rapamycin
NeuN	neuronal nuclei (neuronal marker)
NF-κB	nuclear factor kappa B
NIA	National Institute on Aging
NLRP3	NOD-, LRR- and pyrin domain-containing protein 3
NFT	neurofibrillary tangle
PD	Parkinson’s disease
PET	positron emission tomography
PI3K	phosphoinositide 3-kinase
PPG	preproglucagonergic
RAGE	receptor for advanced glycation end-products
ROS	reactive oxygen species
SH-SY5Y	human neuroblastoma cell line
SOD	superoxide dismutase
STAT	signal transducer and activator of transcription
STZ	Streptozotocin
T2DM	type 2 diabetes mellitus
TG	Triglycerides
TGF-β	transforming growth factor beta
TLR4	Toll-like receptor 4
TNF-α	tumor necrosis factor-alpha
TSH	thyroid-stimulating hormone
VEGF	vascular endothelial growth factor
